# Spin Transport Modulation of 2D Fe_3_O_4_ Nanosheets Driven by Verwey Phase Transition

**DOI:** 10.1002/advs.202405945

**Published:** 2024-09-04

**Authors:** Zhiyan Jia, Mengfan Zhao, Qian Chen, Rong Sun, Lulu Cao, Kun Ye, Tao Zhu, Lixuan Liu, Yuxin Tian, Yi Wang, Jie Du, Fang Zhang, Weiming Lv, FeiFei Ling, Ya Zhai, Yong Jiang, Zhongchang Wang

**Affiliations:** ^1^ Institute of Quantum Materials and Devices School of Materials Science and Engineering Tiangong University Tianjin 300387 China; ^2^ International Iberian Nanotechnology Laboratory (INL) Braga 4715‐330 Portugal; ^3^ Key Laboratory of Quantum Materials and Devices of Ministry of Education School of Physics Southeast University Nanjing 211189 China; ^4^ Key Laboratory of Nanodevices and Applications Suzhou Institute of Nano‐Tech and Nano‐Bionics CAS Suzhou 215123 China; ^5^ School of Electrical and Information Engineering Tianjin University Tianjin 300072 China; ^6^ Hebei Technology Innovation Center of Phase Change Thermal Management of Data Center Hebei University of Water Resources and Electric Engineering Cangzhou 061001 China; ^7^ School of Chemistry Beihang University Beijing 100191 China

**Keywords:** anomalous Hall effect, anisotropic magnetoresistance, Fe3O4, spin mixing conductance, spin hall magnetoresistance

## Abstract

Realizing spin transport between heavy metal and two‐dimensional (2D) magnetic materials at high Curie temperature (*T*_C_) is crucial to advanced spintronic information storage technology. Here, environmentally stable 2D nonlayered Fe_3_O_4_ nanosheets are successfully synthesized using a reproducible process and found that they exhibit vortex magnetic domains at room temperature. A Verwey phase transition temperature (*T*_V_) of ≈110 K is identified for ≈3 nm thick nanosheet through Raman characterization and spin Hall device measurement of the Pt/Fe_3_O_4_ bilayer. The anisotropic magnetoresistance ratio decreases near *T*_V_, while both the spin Hall magnetoresistance ratio and spin mixing conductance (*G_r_*) increase at *T*_V_. As the temperature approaches 112 K, the anomalous Hall effect ratio tends to become zero. The maximum *G_r_* reaches ≈5 × 10^15^ Ω^−1^m^−2^ due to the clean and flat interface between Pt and 2D nanosheet. The observed spin transport behavior in Pt/Fe_3_O_4_ spin Hall devices indicates that 2D Fe_3_O_4_ nanosheets possess potential for high‐power micro spintronic storage devices applications.

## Graphical abstract

The vortex‐state magnetic domains are reported in 2D nonlayered Fe_3_O_4_ nanosheets at room temperature. The Verwey phase transition temperature (*T*_V_) is determined to be ≈110 K for the ≈3 nm thick nanosheet. The anisotropic magnetoresistance ratio decreases near *T*_V_, yet both the spin Hall magnetoresistance ratio and spin mixing conductance (*G*_r_) exhibit an increase at *T*_V_. It is also found that the anomalous Hall effect ratio tends to approach zero as temperature is close to 112 K. The maximum *G*_r_ reaches ≈5 × 10^15^ Ω^−1^m^−2^, which is attributed to the clean and flat interface between Pt and 2D nanosheet. These findings indicate potential applications of 2D Fe_3_O_4_ nanosheets in high‐power microspintronic storage devices.

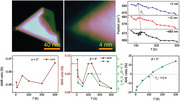

## 1 Introduction

The investigation of spin transport across interfaces in heavy metal/magnetic material bilayer structures is crucial to its potential application in spintronic information storage devices.^[^ ^1^, ^2^ ^]^ However, the size and power optimization of these storage devices depend heavily on advancements in magnetic materials.^[^ ^3^, ^4^ ^]^ The successful doping of magnetic V element in transition mental dichalcogenide semiconductors has previously been effective in producing stable, room‐temperature magnetic nanosheets.^[^ ^5^, ^6^, ^7^, ^8^ ^]^ This also fosters the study of intrinsic magnetic two‐dimensional (2D) layered materials, such as Cr_2_Ge_2_Te_6_,^[^ ^9^ ^]^ CrI_3_,^[^ ^10^ ^]^ Fe_3_GeTe_2_,^[^ ^11^ ^]^ Fe_4_GeTe_2_,^[^ ^12^ ^]^ Cr_7_Te_8_,^[^ ^13^ ^]^ MnSiTe_3_,^[^ ^14^ ^]^ and Fe_3_GaTe_2_,^[^ ^15^ ^]^ has sparked research interest in novel spintronic devices like 2D spin–orbit torque and magnetic tunnel junction devices^[^ ^16^, ^17^, ^18^, ^19^ ^]^ where spin transport can be utilized for information storage purpose.^[^ ^20^ ^]^ Nevertheless, most of the 2D layered magnetic materials possess limitations including poor environmental stability or low Curie temperature (*T*_C_), posing a significant challenge to their practical application.^[^ ^21^ ^]^ Recently, 2D nonlayered magnetic materials, such as *ε*–Fe_2_O_3_,^[^ ^22^ ^]^ *γ*–Fe_2_O_3_,^[^ ^23^, ^24^ ^]^ Fe,^[^ ^25^ ^]^ Fe_7_Se_8_,^[^ ^26^ ^]^ CoFe_2_O_4_,^[^ ^27^ ^]^ Cr_5_Te_8_,^[^ ^28^ ^]^ Mn_0.14_Cr_0.86_Te,^[^ ^29^ ^]^ and Fe_3_O_4_^[^ ^30^ ^]^ with high *T*_C_ have captured a lot of attention. Among them, 2D Fe_3_O_4_ is a classic ferrimagnetic oxide with an inverse spinel structure that exhibits half metallicity at room temperature^[^ ^31^ ^]^ and shows excellent air stability and 100% spin polarization.^[^ ^32^ ^]^ It is thus an ideal platform for investigating 2D magnetism and spin devices.^[^ ^33^ ^]^ Additionally, Fe_3_O_4_ possesses a distinctive crystallographic feature known as the Verwey phase transition at low temperature (*T*_V_), which has been extensively reported in Fe_3_O_4_ films,^[^ ^31^, ^34^, ^35^ ^]^ similar to the famous Morin phase transition observed in *α*‐Fe_2_O_3_.^[^ ^36^ ^]^ The magnetoelectric features of Fe_3_O_4_ are influenced by the Verwey phase transition within its structure, including metal–insulator transition at *T*_V_.^[^ ^33^ ^]^ The underlying physical mechanism of this transition remains a long–standing puzzle, despite Verwey's initial hypothesis of charge ordering of Fe^2+^ and Fe^3+^ ions on octahedral sites (B sites).^[^ ^31^, ^37^ ^]^ Previous investigations on magnetoresistance (MR) and the spin anomalous Hall effect (AHE) of heavy metal/Fe_3_O_4_ films have revealed a large spin mixing conductance (*G_r_*), demonstrating its potential application.^[^ ^38^, ^39^ ^]^ Therefore, a comprehensive study of *T*_V_, MR, AHE, and *G_r_* of Fe_3_O_4_ nanosheets is crucial for their utilization in spintronic information storage devices, despite that such research is to date scarce.

Here, we synthesized 2D Fe_3_O_4_ nanosheets using the chemical vapor deposition (CVD) method and observed vortex state magnetic domains at room temperature. The *T*_V_ of Fe_3_O_4_ nanosheets is found to be ≈124, ≈120, and ≈110 K for the nanosheets with thicknesses of ≈388, ≈12, and ≈3 nm, respectively, indicating a positive correlation between *T*_V_ and thickness. Through anisotropic magnetoresistance (AMR), spin Hall magnetoresistance (SMR), and AHE investigations, we observe significantly large MR and *G_r_* (≈5 × 10^15^ Ω^−1^m^−2^) in Pt/Fe_3_O_4_ spin Hall devices due to the flat and clean interface between the heavy metal layer and 2D nanosheets. Additionally, the spin transport properties of the Pt/Fe_3_O_4_ spin Hall devices are found to be influenced by the Verwey phase transitions of Fe_3_O_4_ at low temperature. These findings on the 2D Fe_3_O_4_‐based spin Hall devices with a well‐defined interface demonstrate immense potential for next‐generation spintronic devices.

## 2 Results and Discussion

### 2.1 Innovative Concepts and Design

The interfaces of 2D spintronics devices play a crucial role in spin transport with the *G_r_* serving as a key parameter for assessing interface quality. Among the known 2D magnetic materials, Fe_3_O_4_ nanosheets are recognized as one of the excellent room‐temperature magnetic materials, yet the evaluation of their interface with other contact materials still poses a significant challenge. In this study, we employed the CVD to grow reproducible and stable 2D Fe_3_O_4_ nanosheets, followed by the deposition of Hall bar shaped Pt strips to quantitatively characterize their interfacial quality using spin transport methods (e.g., obtaining AMR, SMR, AHE, and *G_r_*). Furthermore, we explore the correlation between the Verwey phase transition features of Fe_3_O_4_ and spin transport to provide in‐depth understanding of the underlying mechanism.

### 2.2 Synthesis of 2D Fe_3_O_4_ Nanosheets

**Figure 1:**
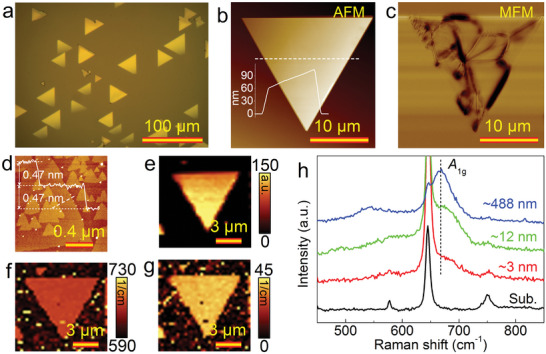
Characterization of 2D Fe_3_O_4_ nanosheets. a–c) Optical, AFM, and MFM images of Fe_3_O_4_ nanosheets on sapphire. The inset in (b) gives height profile taken along the dashed line. d) High–magnification AFM image of the surface of the 2D Fe_3_O_4_ nanosheet in (b). e–g) Intensity, position, and FWHM signal mapping images of the Raman peak at ∼670 cm^−1^. h) Raman spectra of the sapphire substrate and as–grown Fe_3_O_4_ nanosheets with thicknesses of ≈3, ≈12, and ≈488 nm, respectively.

The 2D Fe_3_O_4_ nanosheets were synthesized on sapphire using the CVD method, as depicted in **Figures** **1**a and S1a (Supporting Information). Surface morphology and magnetic structure of Fe_3_O_4_ nanosheets were analyzed using AFM and MFM (Figure. 1b–d and Figure S1b–e, Supporting Information). Vortex–state magnetic domains were observed at room temperature, accompanied by lamellar–like steps above the nanosheet (Figure 1d). Each individual lamellar step of Fe_3_O_4_ exhibited a thickness of ≈0.47 nm, corresponding to a height equivalent to 1/3 unit–cell (as illustrated in Figure S2, Supporting Information). Experimentally, we repetitively synthesized ultrathin Fe_3_O_4_ nanosheets for Raman and spin transport studies (Figure S1b‐e, Supporting Information). As illustrated in Figure S1b–e (Supporting Information), some ultrathin Fe_3_O_4_ nanosheets were synthesized for Raman and spin transport studies. X‐ray diffraction (XRD) analysis was also conducted to characterize the Fe_3_O_4_ nanosheets (Figure S3, Supporting Information).^[^ ^40^, ^41^ ^]^ Further analysis of the Fe_3_O_4_ Raman peak at ∼670 cm^−1^ revealed high uniformity of peak intensity, position, and full width at half maximum (FWHM) across the entire triangular Fe_3_O_4_ nanosheet (Figures 1e–g). Furthermore, thickness dependent Raman spectra (≈3, ≈12, and ≈488 nm) confirmed the dominant presence of the ≈670 cm^−1^ peak originating from *A*_1g_ mode vibrations characteristic of the Fe_3_O_4_ crystal. These results confirm successful synthesis of uniform Fe_3_O_4_ nanosheets. It is worth noting that while the intensity of the *A*_1g_ Raman peak is positive correlation with nanosheet thickness as previously reported, its specific position and width are less influenced by variations in thickness,^[^ ^30^ ^]^ suggesting that the crystal structure of Fe_3_O_4_ remains stable with the variation of thickness. In addition, the AFM, MFM, and Raman signals of Fe_3_O_4_ nanosheets with different thicknesses were stable and reproducible when they were placed in air for two years of aging (Figures S4 and S5, Supporting Information), confirming their chemical stability. Such Fe_3_O_4_ nanosheets can be maintained in both clean‐room environment and annealing in vacuum, which demonstrates their superb chemical stability.^[^ ^33^ ^]^

### 2.3 Work Function of 2D Fe_3_O_4_ Nanosheet

**Figure 2:**
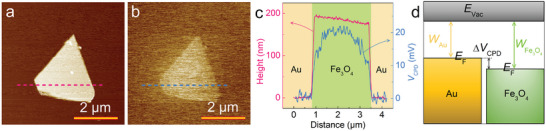
KPFM Characterization of 2D Fe_3_O_4_ nanosheets. a,b) AFM (a) and KPFM (b) images acquired within an area encompassing a 2D Fe_3_O_4_ nanosheet. c) Line profiles of height (red) and *V*_CPD_ (blue) extracted along the dashed lines indicated in the corresponding AFM and KPFM images in (a) and (b), respectively. Both curves are baseline‐corrected. d) Energy band diagram illustrating the junction between 2D Fe_3_O_4_ and Au. The *E*_Vac_ represents the reference vacuum level, $W_{Fe_{3}O_{4}\;\left(\mathrm{or}\;\mathrm{Au}\right)}$ denotes the work function, and Δ*V*_CPD_ donotes the Fermi difference between Fe_3_O_4_ and Au film.

The accurate determination of work function (*W*) of a material is crucial to device fabrication. To investigate the work function ($W_{Fe_{3}O_{4}}$) of Fe_3_O_4_ nanosheets, we transferred them onto a gold film with a known work function (*W*_Au_ = ≈5.1 eV).^[^ ^42^ ^]^ **Figure** **2**b illustrates the kelvin probe force microscopy (KPFM) mapping image corresponding to the AFM topography scan of a 2D Fe_3_O_4_ nanosheet (Figure 2a). The topographic and contact potential contours of these nanosheets are summarized in Figure 2c, which have been background‐subtracted and naturally establish a correlation between the nanosheet and surface contact potential (*V*_CPD_). It should be noted that *V*_CPD_ can be obtained using the following equation:

(1) $$eV_{\mathrm{CPD}}=W_{\mathrm{tip}}-W_{\mathrm{sample}}$$

where *W*_tip_ and *W*_sample_ represent the respective work functions of the tip and sample, and *e* denotes the elementary charge in absolute value.

The contact potential difference (or Fermi difference, Δ*V*_CPD_) between the Fe_3_O_4_ nanosheet and Au film is determined by the equation:

(2) $$\begin{array}{c} \Delta V_{\mathrm{CPD}}=V_{\mathrm{CPD}(Fe_{3}O_{4})}-V_{\mathrm{CPD}(\mathrm{Au})}=E_{F(Fe_{3}O_{4})}-E_{F(\mathrm{Au})}=W_{Fe_{3}O_{4}}-W_{\mathrm{Au}} \end{array}$$

where ${V_{\mathrm{CPD}}}_{\left(Fe_{3}O_{4}\right)}$ and *V*_CPD(Au)_ represent distinct brightness contrast due to the disparity in work function between $W_{Fe_{3}O_{4}}$ and *W*_Au_, during the KPFM measurement.^[^ ^42^ ^]^ The positive ∆*V*_CPD_ (≈20 mV) indicates that the Fermi energy level of Fe_3_O_4_ is lower than the vacuum energy level of Au, as shown in Figure 2d.^[^ ^43^, ^44^, ^45^ ^]^ This finding not only provides valuable insights into designing Fe_3_O_4_‐based devices but also validates our selection of Pt with a high work function (≈5.41 eV) as the contact electrode in this study.^[^ ^46^, ^47^ ^]^

### 2.4 Atomic–Scale Structure

**Figure 3:**
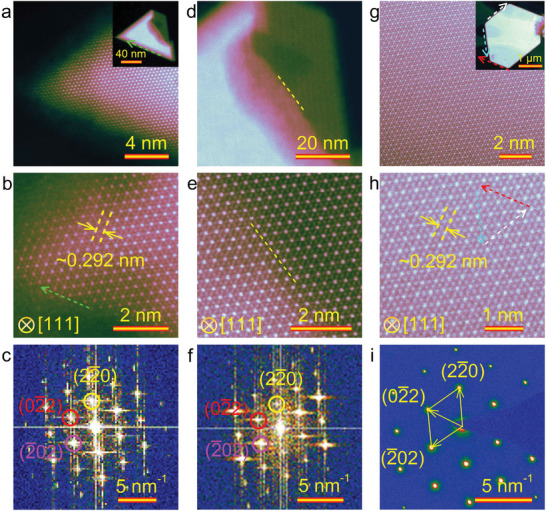
Structural characterization of the triangular and hexagonal 2D Fe_3_O_4_ nanosheets. a) Low– and b) high–magnification HAADF STEM images of an edge of a triangular 2D Fe_3_O_4_ nanosheet. The inset in (a) is a STEM image of the broken triangular Fe_3_O_4_ nanosheet. c) Corresponding FFT patterns of the triangular nanosheet in (a). d) STEM image of the broken part of the triangular nanosheet. e) HAADF STEM image of the area in the transitional step. f) Corresponding FFT patterns of the STEM image for the transitional area in (e). g) Low– and h) high–magnification HAADF STEM images of a hexagonal 2D Fe_3_O_4_ nanosheet. The inset in (g) shows STEM image of the hexagonal Fe_3_O_4_ nanosheet. i) Corresponding SAED pattern of the hexagonal Fe_3_O_4_ nanosheet in (g).

The bulk Fe_3_O_4_ possesses an inverse spinel cubic structure, with iron ions occupy interstitial tetrahedral sites (A sites, Fe^3+^) and octahedral sites (B sites, eight Fe^2+^, and eight Fe^3+^ ions), while oxygen ions form a face‐centered cubic (FCC) packing structure.^[^ ^31^ ^]^ **Figure** **3**a,b shows high‐angle annular dark‐field scanning transmission electron microscopy (HAADF STEM) images revealing the atomic configuration of iron with zigzag termination edges, as indicated by green arrows. The estimated interplanar spacing for the (2$\bar{2}$0) plane is determined to be 0.292 nm, consistent with the top view of Fe_3_O_4_ along the [111] zone axis shown in Figure 3b. Additionally, Figure 3c displays the corresponding fast Fourier transform (FFT) pattern derived from the HAADF STEM image, which exhibits a set of sixfold symmetry diffraction spots supporting the synthesis of single‐crystalline 2D Fe_3_O_4_. Furthermore, a distinct step position is observed in the triangular Fe_3_O_4_ nanosheet (Figure 3d), indicating an ordered arrangement of Fe and O atoms along the step marked with a white dashed line in Figure 3e. This observation is further supported by the FFT pattern showing one set of sixfold spots, as depicted in Figure 3f. These findings provide compelling evidence that the stepped 2D Fe_3_O_4_ is epitaxially grown, consistent with its crystal structure shown in Figure S1 (Supporting Information). Additionally, a few hexagonal Fe_3_O_4_ nanosheets are obtained during growth (Figure 3g). A comparison between these images and atomic arrangement of the three neighboring edges highlighted by dashed lines in red, white, and cyan in Figure 3g,h reveals that Fe atoms exhibit a zigzag pattern similar to that observed in the triangular nanosheet. The selected area electron diffraction (SAED) image shown in Figure 3i confirms that both triangular and hexagonal Fe_3_O_4_ nanosheets possess identical crystal structures through matching diffraction spots with face spacing (≈0.292 nm) corresponding to the (2$\bar{2}$0).

### 2.5 Raman Characterizations

**Figure 4:**
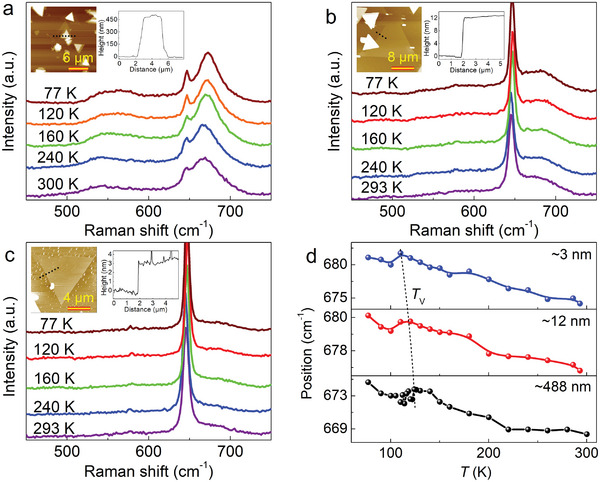
Characterization of the Verway phase transition temperature of 2D Fe_3_O_4_ nanosheets with different thicknesses. a) Raman spectra of ≈488 nm Fe_3_O_4_ nanosheet obtained at temperature ranging from 77 to 300 K. b) Raman spectra of ≈12 nm Fe_3_O_4_ nanosheet obtained at various low temperatures. c) Raman spectra of ≈3 nm Fe_3_O_4_ nanosheet obtained at different temperatures. The inset shows AFM image and height line profiles of the corresponding nanosheets. d) Relative Raman peak position offset–temperature curves for the *A*_1g_ mode. As temperature decreases, a Verway phase transition was observed in the Fe_3_O_4_ nanosheets.

It remains challenging to detect the Verwey phase transition temperature (*T*_V_) of Fe_3_O_4_ films with a thickness of <10 nm.^[^ ^48^, ^49^, ^50^ ^]^ To investigate the Verwey phase transition process of 2D Fe_3_O_4_ nanosheets with varying thickness, Raman spectroscopy measurements were conducted in a temperature range from 77 to 300 K (Figure S6, Supporting Information). Figure 3a–c depicts the Raman spectra of three nanosheets with a thickness of ≈488, ≈12, and ≈3 nm, respectively. **Figure** **4**a–c shows AFM images and surface line profiles of the nanosheets, revealing that as temperature decreases, both the deconvoluted FWHM and integral intensity of Raman peak *A*_1g_ decrease, despite of the irregular fluctuation (Figure S7, Supporting Information), indicating a reasonable fitting process. Figure 4d illustrates that the deconvoluted peak position of Raman peak *A*_1g_ undergoes a blue shift upon decreasing temperature. Notably, at low temperatures, there is an abrupt jump in the peak position due to the occurrence of Verwey phase transition. Based on this observation, it can be inferred that *T*_V_ of the nanosheets with thicknesses of ≈388, ≈12, and ≈3 nm is ≈124, ≈120, and ≈110 K, respectively. This positive correlation between *T*_V_ and thickness indicates that internal defects and stress originating from substrate interactions increase as thickness decreases, consistent with previous reports.^[^ ^49^, ^50^ ^]^ The determination of *T*_V_ for the 3 and 12 nm‐thick nanosheets play a crucial role in interpreting their structure and phase transitions, enabling in–depth understanding of spin transport variation within Fe_3_O_4_‐based spin devices under different temperature.

### 2.6 Spin Transport of Pt/Fe_3_O_4_ Spin Hall Device

**Figure 5:**
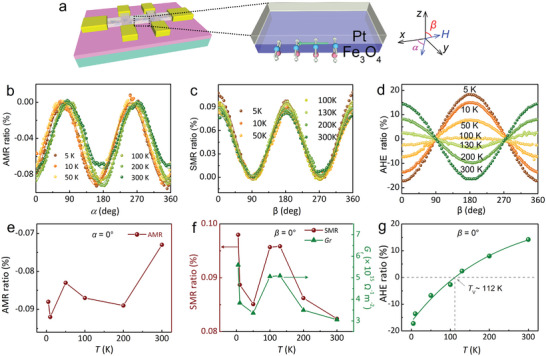
Temperature‐dependent electrical transport for a Pt/Fe_3_O_4_ Hall device (≈3 nm). a) Sketch of the Pt/Fe_3_O_4_ bilayered Hall bar device. b) AMR ratio versus the angle *α* in *xy* plane at different temperature. c) SMR ratio and d) AHE ratio as a function of the angle *β* between magnetic field and *z* direction in the *yz* plane under different temperatures. The AMR ratio, SMR ratio, and AHE ratio are extracted from [*R_xx_* − *R_xx_* (*α* = 90°)]/*R_xx_* (*α* = 90°), [*R_xx_* − *R_xx_* (*β* = 90°)]/*R_xx_* (*β* = 90°), and [*R_xy_* − *R_xy_* (*β* = 90°)]/*R_xy_* (*β* = 90°), respectively. e) The AMR ratio, f) SMR ratio and *G*_r_, and g) AHE ratio as a function of temperature, when the *α* or *β* is zero.

To elucidate the impact of Verwey phase transition on spin transport behavior in 2D Fe_3_O_4_ nanosheets, we fabricated a Pt/Fe_3_O_4_ spin Hall device (**Figures** **5**a and S8a, Supporting Information), where thickness of the Fe_3_O_4_ nanosheets mainly ranges below 10 nm (Figure S8b,c, Supporting Information). A robust spin–transporting channel is formed by employing a heavy Pt film with pronounced spin–orbit coupling. We conducted systematic study of magnetoresistance (*R*_xx_) and anomalous Hall magnetoresistance (*R*_xy_) of the device under a range of temperature and magnetic field conditions (Figure S9, Supporting Information). The magnetoresistance (MR) and anomalous Hall loops were saturated at an external magnetic field of ≈9 T, with a change in polarity observed at ≈100 K. To further probe the evolutionary patterns of spin transport, we investigated the angular dependence of *R*_xx_ and *R*_xy_. Specifically, *α*‐scans (rotating the magnetic field *H* in the *xy* plane) and *β*‐scans (rotating *H* in the *yz* plane) were performed to collect *R*_xx_ and *R*_xy_ (Figure 5a), respectively. These measurements enable the determination of the AMR ratio, SMR ratio, and AHE ratio as a function of the angles *α*, *β*, and *β* at various temperatures (Figure 5b‐d). The magnetic field strength is maintained at 9 T for all measurements. The AMR ratio, SMR ratio, and AHE ratio were calculated using specific equations:^[^ ^51^, ^52^ ^]^

(3) $$\mathrm{AMR}\;\mathrm{ratio}\;=\;\frac{R_{xx}-R_{xx}\left(\alpha \;=\;90^{\circ }\right)}{R_{xx}\left(\alpha \;=\;90^{\circ }\right)}$$

(4) $$\mathrm{SMR}\;\mathrm{ratio}\;=\;\frac{R_{xx}-R_{xx}\left(\beta \;=\;90^{\circ }\right)}{R_{xx}\left(\beta \;=\;90^{\circ }\right)}$$

(5) $$\mathrm{AHE}\;\mathrm{ratio}\;=\;\frac{R_{xy}-R_{xy}\left(\beta \;=\;90^{\circ }\right)}{R_{xy}\left(\beta \;=\;90^{\circ }\right)}$$

The AMR and SMR ratios are derived from *R_xx_* by rotating *α* in *xy* plane and *β* in *yz* plane respectively. Similarly, the AHE ratio is obtained from the *R_xy_* by rotating *β* in the *yz* plane.

Angular dependent resistance analysis reveals a strong correlation between temperature and the extracted signals of AMR, SMR, and AHE. The features related to AMR are observed in the temperature range of 5–300 K and exhibit consistent angular dependence characterized by a twofold symmetry. Notably, visible MR is identified when the external magnetic field rotates in the *xy* plane (*α*–scan) within the temperature range from 5 to 300 K. Figure 5e shows that as the temperature crosses through *T*_V_, there is a decreasing trend in the AMR ratio. The observed negative AMR in Fe_3_O_4_ results from the dominant *s*‐*d* scattering process, where the density of states *D_σ_* and resistivity *ρ_σ_* conditions (*σ* = ↑ or ↓) lead to *D*_↑_^(^ *^d^* ^)^<*D*_↓_^(^ *^d^* ^)^ and *ρ*_↑_>*ρ*_↓_, respectively.^[^ ^53^ ^]^ These findings reveal that Fe_3_O_4_ is an efficient anisotropic material, albeit that the SMR of nanosheets is scarcely reported.^[^ ^41^, ^54^, ^55^ ^]^ The angle–dependent magnetoresistance measurements were conducted to investigate SMR ratio (Figure 5c). According to the SMR ratio, spin mixing conductance (*G_r_*) at Pt/Fe_3_O_4_ can be obtained by:^[^ ^51^, ^56^ ^]^

(6) $$\mathrm{SMR}\;\mathrm{ratio}\;=\;\theta_{SH}^{2}\frac{\lambda }{d_{N}}\;\frac{2\lambda G_{r}\tan h^{2}\frac{d_{N}}{2\lambda }}{\sigma +2\lambda G_{r}\cot h\frac{d_{N}}{\lambda }}$$

where λ, *θ_SH_*, *d_N_*, and *σ* represent spin diffusion length, spin Hall angle, thickness, and electrical conductivity of the heavy metal Pt film, respectively.^[^ ^51^ ^]^ Here, we use *λ* = 1.5 nm, *θ_SH_* = 1.5 nm and σ = 5 × 10^6^ Ω^−1^m^−1^.^[^ ^57^, ^58^ ^]^ The obtained values at various temperature are presented in Figure 5f. The maximum *G_r_* is ≈5 × 10^15^ Ω^−1^m^−2^, which far exceeds those reported for the sputtered *γ*‐Fe_2_O_3_/NiO/Pt, Pt/*α*‐Fe_2_O_3_ and Pt/Fe_3_O_4_ bilayered heterojunctions due to the clean and flat interface between the 2D nanosheet and heavy metal.^[^ ^38^, ^59^, ^60^ ^]^ Table S1 (Supporting Information) lists the comparison of the relevant parameters from the literatures. Both SMR ratio and *G_r_* are closely dependent on temperature with the enhancement peaks at ∼110 K (Figure 5f), providing evidence for the occurrence of Verwey phase transition at *T*_V_.

The AHE ratio related to *β*–scan shows an opposite sign from 130 to 100 K in Figure 5d, corresponding to the Verwey phase transition. As the temperature decreases to ≈112 K, the AHE ratio approaches zero, providing evidence that *T*_V_ occurs at ≈112 K (Figure 5e). This finding is consistent with low–temperature Raman characterization of thin nanosheets. We also utilized 2D Fe_3_O_4_ nanosheets to fabricate a heavy metal Pt/Fe_3_O_4_ spin Hall device, leading to large MR and *G_r_*. It is worthy of noting that temperature plays a crucial role in spin transport, specifically AMR, SMR, and AHE alter across *T*_V_ as supported by Raman characterization at low temperature.

## 3 Conclusion

We have successfully synthesized 2D nonlayered Fe_3_O_4_ nanosheets on sapphire substrates using the reproducible CVD method, and demonstrated that they are environmentally stable for over two years. We provide direct observation of the presence of vortex state magnetic domains at room temperature. The triangular and hexagonal nanosheets display uniform zigzag edges, with a spacing of ≈0.292 nm for the (2$\bar{2}$0) face for Fe_3_O_4_ nanosheets. The *T*_V_ decreases as the thickness of the 2D nanosheets decreases, and the ≈3 and ≈12 nm‐thick nanosheets undergo the Verwey phase transitions at ≈110 and ≈120 K, respectively. As temperature approaches *T*_V_, the AMR ratio decreases, while both SMR ratio and *G*_r_ increase suddenly. Conversely, the AHE ratio exhibits an opposite trend as temperature decreases from 300 to 5 K due to the Verwey phase transition. Notably, AHE ratio approaches zero at ≈112 K, confirming that *T*_V_ for thinner layers is close to 110 K. Furthermore, the Pt/Fe_3_O_4_ spin Hall device demonstrates a large *G_r_* (≈5 × 10^15^ Ω^−1^m^−2^), which is attributed to its clean and flat interface between heavy metal layer and 2D nanosheets. This detection of *T*_V_ in ultrathin Fe_3_O_4_ nanosheets highlights their significantly tunable spin transport properties in nanostructured spin Hall devices, offering promising alternatives for next‐generation magnetic and spintronic applications.

## 4 Experimental Section

### Growth of 2D Fe_3_O_4_ Nanosheets by CVD

Synthesis of Fe_3_O_4_ nanosheets involves the use of a mixture of Fe (0.09 g, 99.9%, Alfa Aesar) and NaCl (0.03 g, 99.9%, Alfa Aesar) powder as precursor and Al_2_O_3_ as growth substrate. The growth substrate was positioned upside down in the center of high‐temperature region. Successful synthesis of 2D nanosheets on sapphire substrates was accomplished within a duration of 30 min at 800 °C (Figure S1, Supporting Information).

### Structural Characterization

The Optical, altitudinal, and magnetic images were acquired using optical microscope (OM, PSM–1000, Moti), atomic force microscope (AFM, Dimension Icon, Bruker), and magnetic force microscope (MFM, Dimension Icon, Bruker), respectively. Raman results were obtained utilizing a confocal Raman microscope (Raman, LabRAM HR Evolution, Horiba) equipped with a low‐temperature chamber (HFS600E–PB4, Linkam). All STEM images were captured using a double‐corrected transmission electron microscope (Titan G2 Cubed Themis, FEI) operated at 200 kV with a probe convergence angle of 21.4 mrad. Surface potential images were acquired by employing a Kelvin probe force microscope (KPFM, Dimension Icon, Bruker). XRD measurements were carried out on a PANalytical X'Pert MPD equipped with an X'Celerator detector and secondary monochromator (Cu Kα λ = 0.154 nm). All the aging experiments (AFM and Raman mapping images) were conducted with the samples exposed in ambient air environment (Figures S4 and S5, Supporting Information).

### Hall Device Fabrication and Spin Hall Transport Measurements

Spin Hall devices were fabricated using standard photolithography techniques, with the assistance of electron beam lithography (eLINE Plus, Raith). A 4 nm Pt Hall bar was patterned through sputtering, exposure, and etching processes. Subsequently, Ti/Au layers (10 nm/60 nm) were deposited by electron beam evaporation under a pressure of 3 × 10^−6^ Torr. Images of the device are presented in Figure S8 (Supporting Information). Magnetoresistance and Hall resistance measurements were conducted using a homemade magnetoelectricity system that allowed for rotation of magnetic field in both the *xy* plane (*α*–scan) and *yz* plane (*β*–scan). The obtained data enabled calculation of the AMR ratio, SMR ratio, and AHE ratio based on the respective angles *α*, *β*, and *β*, respectively. All measurements were performed over a temperature range from 5 to 300 K.

## Conflict of Interest

The authors declare no conflict of interest.

## Author Contributions

Z.Y.J. and M.F.Z. contributed equally to this work. Z.C.W., Y.J., Y.Z., and Q.C. supervised and led the project. Z. Y. J. and M. F. Z. synthesized the 2D Fe_3_O_4_ nanosheets. R.S., K.Y., L.X.L., Y.X.T., Y.W., F.Z., T.Z., J.D., and F.F.L. conducted comprehensive AFM, MFM, Raman, and STEM characterizations. Q.C., L.L.C., and W.M.L. fabricated spin Hall devices and carried out magnetoelectricity measurements. All authors analyzed the data and contributed to manuscript preparation.

## Supporting information

Supporting Information

## Data Availability

The data that support the findings of this study are available from the corresponding author upon reasonable request.
