# Supplementary material for: Spin Transport Modulation of 2D Fe3O4 Nanosheets Driven by Verwey Phase Transition
Source: Adv Sci (Weinh). 2024 Sep 4;11(41):2405945. doi: 10.1002/advs.202405945 (PMC11538658; doi:10.1002/advs.202405945)
Supplement: Supplementary file 1 — Supporting Information [file ADVS-11-2405945-s001.docx]

**Supporting Information**

Spin Transport Modulation of Two-dimensional Fe_3_O_4_ Nanosheets Driven by Verwey Phase Transition

*Zhiyan Jia^1,2#^, Mengfan Zhao^1#^, Qian Chen^3,4*^, Rong Sun^2^, Lulu Cao^3^, Kun Ye^1^, Tao Zhu^1^, Lixuan Liu^1^, Yuxin Tian^1^, Yi Wang^1^, Jie Du^1^, Fang Zhang^1^, Weiming Lv^4^, FeiFei Ling^5,7^, Ya Zhai^3^, Yong Jiang^1*^, Zhongchang Wang^2,6*^*


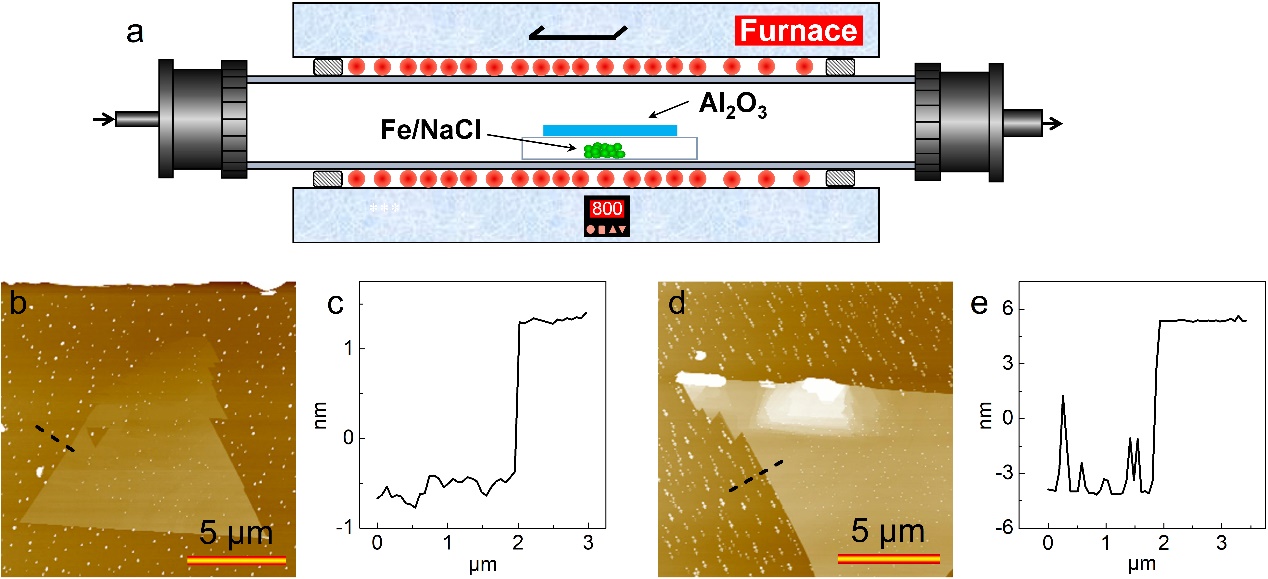


**Figure S1. The CVD system and images of ultrathin Fe_3_O_4_ nanosheets.** a) Schematic diagram of the CVD setup used for the experiment. b) AFM image and (c) line profile of a ~2 nm Fe_3_O_4_ nanosheet. d) AFM image and (e) line profile of a ~9 nm Fe_3_O_4_ nanosheet.


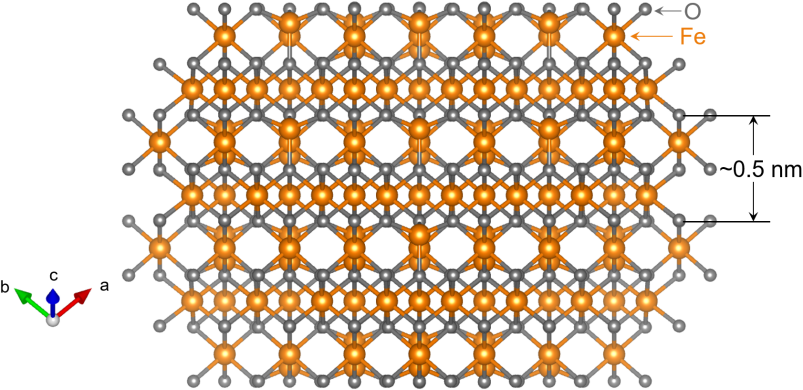


**Figure S2. Atomic structure of the cross-section of a 2D Fe_3_O_4_ nanosheet.**


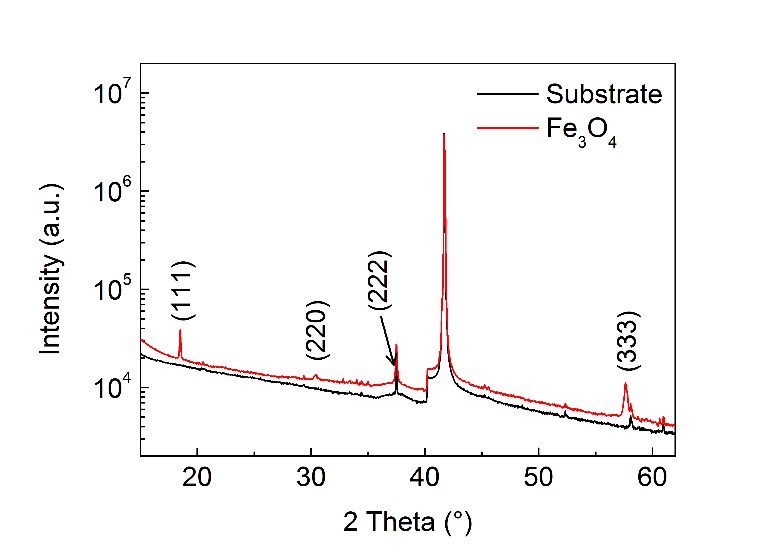


**Figure S3.** XRD patterns of the Fe_3_O_4_ nanosheets and Al_2_O_3_ substrate, respectively.


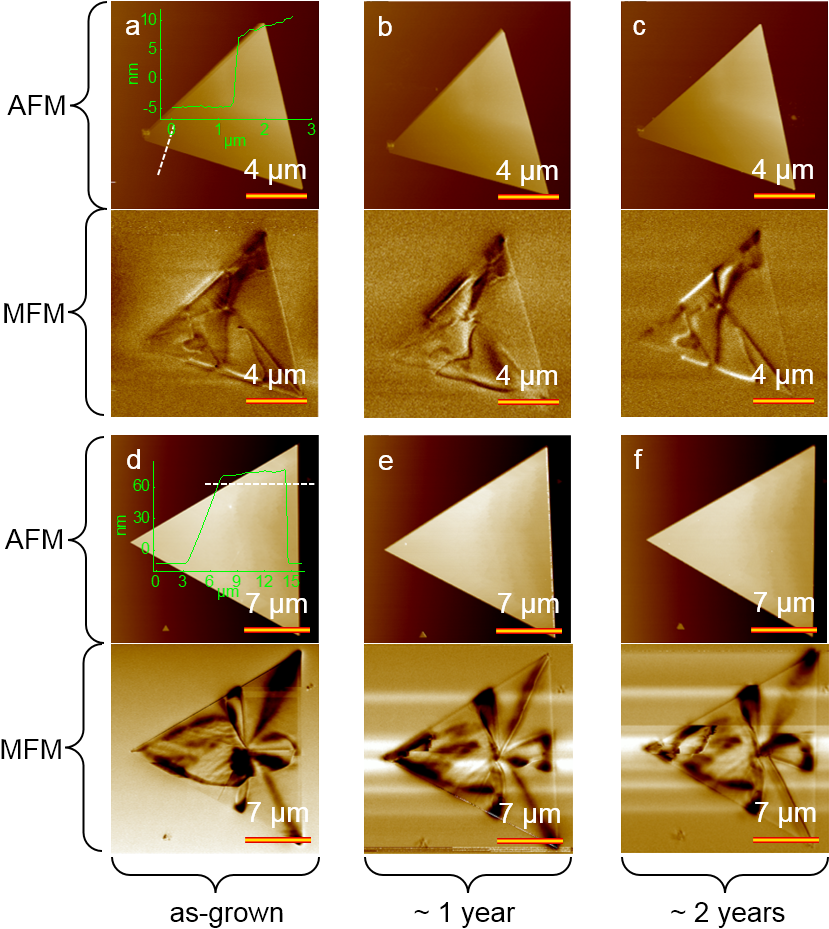


**Figure S4.** **AFM and MFM image analysis of Fe_3_O_4_ nanosheets subjected to aging experiments.** AFM (upper) and MFM (lower) images of Fe_3_O_4_ nanosheets (~13 nm) depicting their condition (a) as-grown, (b) after one year of storage, and (c) after two years of storage. AFM (upper) and MFM (lower) images of Fe_3_O_4_ nanosheets (~85 nm) depicting their condition (d) as-grown, (e) after one year of storage, and (f) after two years of storage.


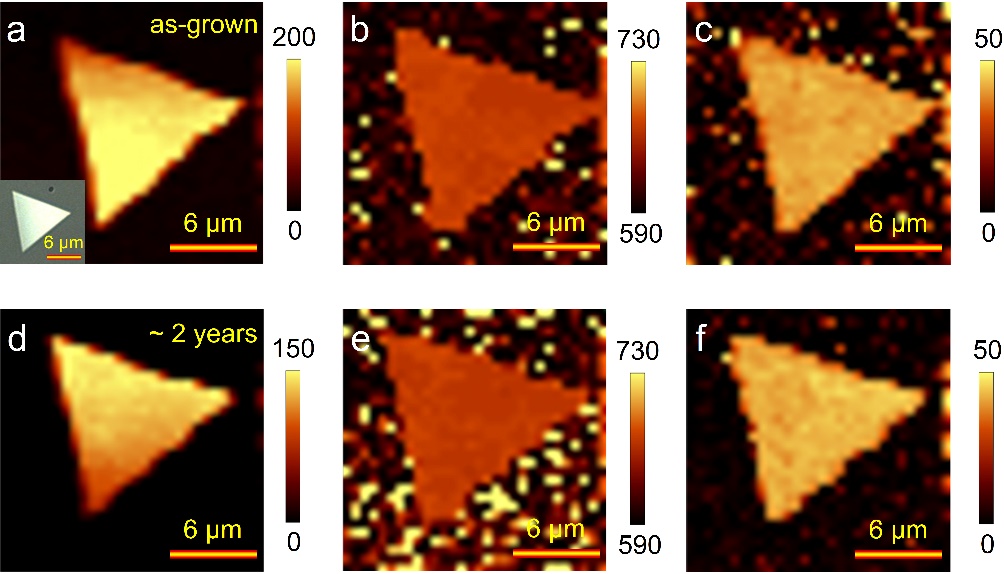


**Figure S5.** **Raman characterizations after ageing.** a-c) Intensity (a), position (b), and width (c) mapping images of the Raman peak at ~670 cm^−1^ for an as-grown 2D Fe_3_O_4_ nanosheet. Inset is the optical image of the nanosheet. d-f) Mapping images of Raman peak intensity (d), position (e), and width (f) at ~670 cm^−1^ for the Fe_3_O_4_ nanosheet after 2 years of aging.


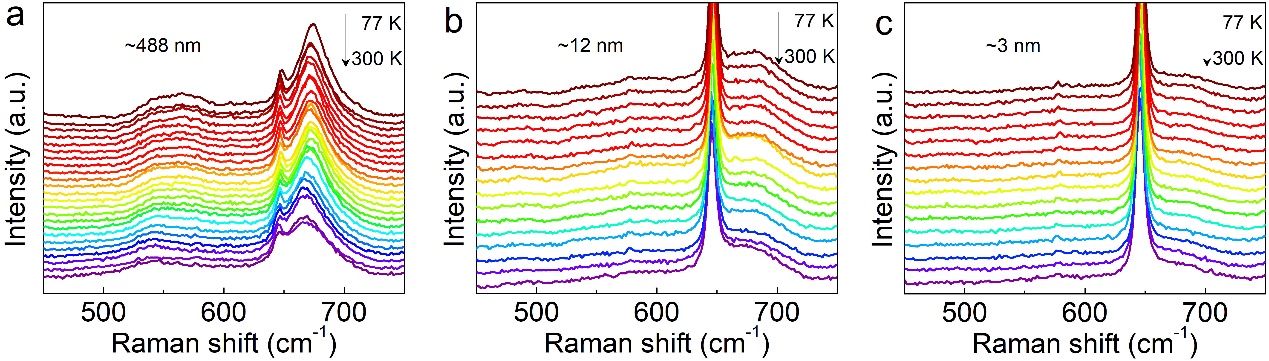


**Figure S6. Raman characterizations of Fe_3_O_4_ nanosheets with different thicknesses.** a) Raman spectra of ~488 nm Fe_3_O_4_ nanosheets at various temperatures. b) Raman spectra were collected ranging from 77 K to 300 K for ~12 nm Fe_3_O_4_ nanosheets. c) Raman spectra of ~3 nm Fe_3_O_4_ nanosheets at various temperatures.


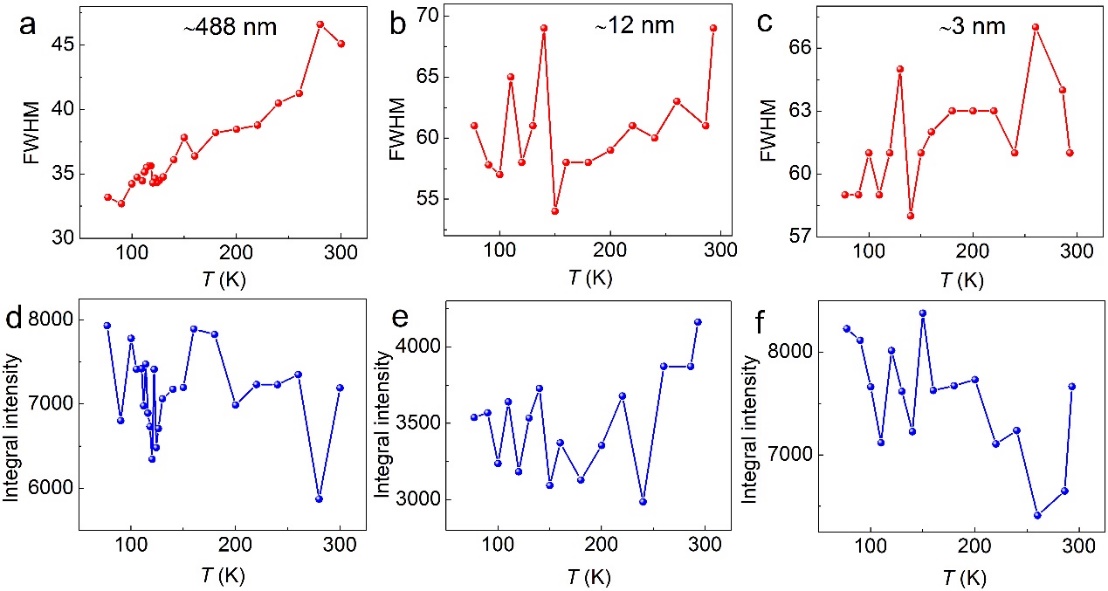


**Figure S7. Raman characterizations of 2D Fe_3_O_4_ nanosheets with different thicknesses.** a–c) Deconvoluted FWHM of Raman peak *A*_1g_ for Fe_3_O_4_ nanosheets ranging from ~488 nm to ~3 nm. d–f) Integral intensity for the *A*_1g_ Raman peak of Fe_3_O_4_ nanosheets with different thicknesses (~488, ~12, and ~3 nm).


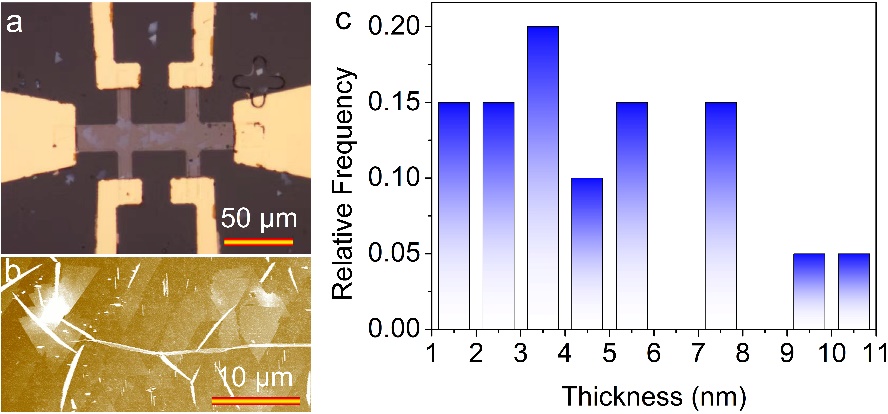


**Figure S8.** a) Optical and (b) AFM images of the 2D Fe_3_O_4_/Pt device. c) The thickness-size distribution of Fe_3_O_4_ nanosheets, the data was obtained by measuring the thickness of 20 nanosheets of the Fe_3_O_4_/Pt device.


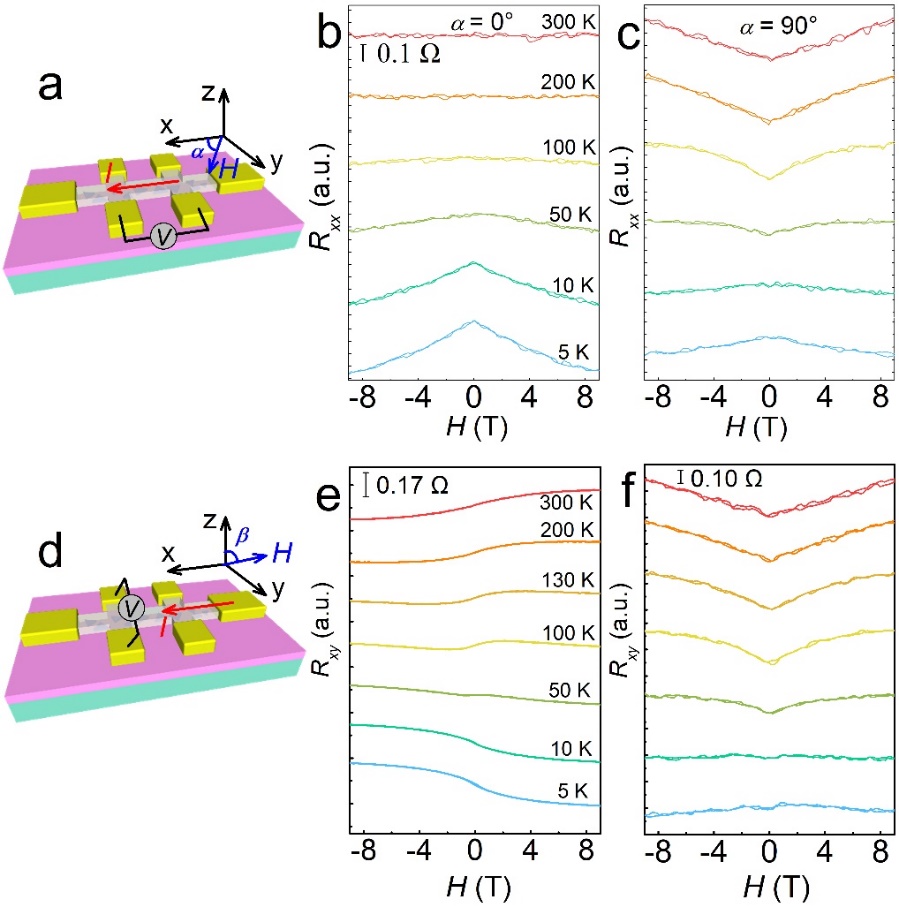


**Figure S9.** a) Illustration of the Pt/Fe_3_O_4_ spin Hall device measurement, where *α* represents the angle between the magnetic field (*H*) and current direction (*x*) in the device. b, c) *R_xx_* dependence on the magnetic field in the temperature range from 5 K to 300 K, which are measured under *α* = 0° and 90°, respectively. d) Illustration of the Pt/Fe_3_O_4_ spin Hall device measurement; *β* represents the angle between the magnetic field (*H*) and perpendicular to the device direction (*y*). e, f) *R_xy_* dependence on the magnetic field in the temperature range from 5 K to 300 K, which are measured under *β* = 0° and 90°, respectively.

**Table S1**. AMR, SMR, and *G_r_* parameters of various spin transport devices.

| **Materials** | **AMR** | **SMR** | ***G_r_* (Ω^-1^m^-2^)** | **Ref.** |
| --- | --- | --- | --- | --- |
| Pt/Fe_3_O_4_ | 8.7×10^-4^ | 9.5×10^-4^ | 5×10^15^ | this work |
| Pt/Fe_3_O_4_ | N/A | 3.7×10^-4^-4.6×10^-4^ | 4.96×10^14^-7.16×10^14^ | [1] |
| *γ*-Fe_2_O_3_/NiO/Pt | N/A | 4×10^-4^ | 1.3×10^14^ | [2] |
| Pt/FePS_3_ | N/A | 3.3×10^-4^ | N/A | [3] |
| Pt/FePS_3_ | N/A | 10×10^-4^ | N/A | [4] |
| Pt/*α*-Fe_2_O_3_ | N/A | 25×10^-4^ | 1.38×10^15^ | [5] |
| Fe_3_O_4_ | 0.5 | N/A | N/A | [6] |

**References**

[1] T. K. H. Pham, M. Ribeiro, J. H. Park, N. J. Lee, K. H. Kang, E. Park, V. Q. Nguyen, A. Michel, C. S. Yoon, S. Cho, T. H. Kim, *Sci. Rep.* **2018**, *8*, 13907.

[2] B.-W. Dong, L. Baldrati, C. Schneider, T. Niizeki, R. Ramos, A. Ross, J. Cramer, E. Saitoh, M. Kläui, *Appl. Phys. Lett.* **2019**, *114*, 102405.

[3] Y. Hui, J. Lu, H. Jiang, W. Lin, C. Dong, K. Dong, Q. He, X. Miao, *J. Phys. D: Appl. Phys.* **2023**, *56*, 075001.

[4] F. Feringa, G. E. W. Bauer, B. J. van Wees, *Phys. Rev. B* **2022**, *105*, 214408.

[5] J. Fischer, M. Althammer, N. Vlietstra, H. Huebl, S. T. B. Goennenwein, R. Gross, S. Geprägs, M. Opel, *Phys. Rev. Appl.* **2020**, *13*, 014019.

[6] F. Lan, R. Zhou, Z. Qian, Y. Chen, L. Xie, *Crystals* **2022**, *12*, 485.
